# Use of narrative medicine to identify key factors for effective doctor–patient relationships in severe asthma

**DOI:** 10.1186/s40248-019-0190-7

**Published:** 2019-09-02

**Authors:** Antonietta Cappuccio, Silvia Napolitano, Francesco Menzella, Guido Pellegrini, Alessandro Policreti, Girolamo Pelaia, Pasquale Alberto Porpiglia, Maria Giulia Marini, SOUND GROUP, Andrea Antonelli, Cesare Arezzo, Stefano Baglioni, Elisa Boni, Alice Bragantini, Domenico Bruzzese, Valeria Caldarelli, Marco Caminati, Lucia Caminiti, Chiara Carraro, Maria Antonietta Ceccon, Fulvia Chieco Bianchi, Anastasia Cirisano, Roberto Cogo, Elisabetta Conte, Enza D’Auria, Saverio Daniele, Dario De Brasi, Giovanna De Castro, Stefano De Luca, Caterina Detoraki, Emanuela Di Palmo, Grazia Fenu, Anna Ferrara, Gaetano Ferrigno, Alessandra Fusi, Annatalia Gaccione, Michela Gandino, Gabriella Guarnieri, Aldo Guerrieri, Corrado Iacoacci, Donato Lacedonia, Mario Lo Schiavo, Rocco Longo, Carla Magazzù, Gabriele Marzo, Michele Mastroberardino, Gian Paolo Mattioli, Liberatore Monaco, Carmen Montera, Marco Morelli, Antonello Nicolini, Pinuccia Omodeo, Gerardo Palmiero, Alessandro Pannofino, Antonio Papa, Francesca Patria, Laura Pini, Stefano Polti, Antonio Pontillo, Mariangela Poppa, Sergio Poto, Oliviero Quercia, Alberto Raie, Vanessa Ronzoni, Yuri Rosati, Antonio Russo, Antonello Salzillo, Marilena Santoro, Francesca Savoia, Anna Simonazzi, Bruno Sposato, Victoria Tourtchenko, Salvatore Tripodi, Alessandro Vatrella, Elisabetta Veronelli

**Affiliations:** 1 Fondazione ISTUD, Milan, Italy; 2 Department of Medical Specialties Pneumology Unit, Arcispedale Santa Maria Nuova- IRCCS, Azienda USL di Reggio Emilia, Reggio Emilia, Italy; 3 Hospital “Città di Sesto San Giovanni” and “E. Bassini”, SC of Paediatric and Neonatology, ASST NordMilano, Sesto San Giovanni, MI Italy; 4 Medical Department Novartis Italia, Origgio, VA Italy; 5 Department of Medicine and Surgery Section of Respiratory Diseases, University “Magna Graecia” of Catanzaro, Catanzaro, Italy

**Keywords:** Narrative medicine, Severe asthma, Medical education and training, Qualitative research

## Abstract

**Background:**

In this project the authors use a narrative medicine (NM) approach to assess the promotion of trust in the relationship between physicians and their asthma patients.

**Methods:**

Following a NM educational course for physicians, a research was carried out in which at least 5 written narratives (parallel charts) for each participating physician were collected and qualitatively analysed according to Bury’s classification and the Grounded Theory.

**Results:**

The results of this study were of speculative and clinical interest. In particular, 66 participants wrote 314 narratives (246 on adult and 68 on paediatric patients). As a result of applying the NM approach, when the relationships remained problematic, many physicians wrote with a moral style about their adult (67%), and paediatric patients (33%) - especially in cases of asthmatic children’s or adolescents’ overprotective or absent families (40%) -. On the contrary, physicians who were able to listen to their patients with empathy (35%) made more shared decisions with patients, even with those they initially had a bad relationship. The used words of welcome, interest and acceptance were promoting patients’ trust that lead to restoring their activities in 45% of cases, according to physicians self-reporting.

**Conclusions:**

These approaches of NM are useful in daily clinical practice, with the goal of improving the quality of life (QOL) of patients with severe asthma, even in cases in which the doctor-patient relationship isn’t initially good.

## Background

### The living with severe asthma

Asthma, a chronic lung disease characterized by airway inflammation, bronchial hyper-responsiveness, and airflow obstruction, affects almost 2.5 million people in Italy. Asthma can occur in intrinsic forms, with unknown pathogenesis, or in extrinsic forms, caused by an allergic response and that occurs in 77–79% of cases [1]. From literature, the most common substances inducing allergic asthma are inhaled allergens like animal dander (skin, saliva), dust mites, cockroach particles, mould and pollen [2]. According to the Global Initiative for Asthma (GINA) Guidelines [3], from 10 to 40% of patients actually suffers from persistent asthmatic breathing [4]; for the development of their therapeutic plan, high dose of inhaled corticosteroids (ICS), beta-agonists (LABA), oral corticosteroids and biologics are recommended [5]. Patients with severe persistent asthma are at a higher risk of negative outcomes, including recurrent and life-threatening exacerbations that significantly affect their quality of life (QOL) [6]. Respiratory Specialists (RS), as pulmonologists, allergists, and paediatricians, often implements a comprehensive strategy for management of uncontrolled symptoms, which can include the assessment of patients’ concordance to treatments, especially when a therapeutic switch is necessary [7]. Nowadays, healthcare is moving from a disease-centred approach, mainly focused on physio-pathological aspects of health, to a patient-centred approach, which emphasizes the illness sphere of patients’ and caregivers’ lives [8]. Furthermore, the awareness of the essential role played by listening and communication skills in determining the patient’s perception of his condition is increasing nowadays. Indeed, understanding illness helps physicians in focusing their care to best address the patient’s real needs. Given all these factors, an effective doctor-patient relationship based on trust is warranted [9]. Indeed, a trusted behaviour, able to lead to shared decision making, is particularly important in the management of severe asthma, as well as in other pathological conditions [10–14].

### Narrative Based Research

Narrative medicine (NM) has been defined as *‘a clinical practice fortified with narrative competence to recognize, absorb, interpret, and respond to stories of the self and others’* [15]. NM is a useful tool to improve patients’ and physicians’ trust and to collect information on the perceived needs of patients and physicians [16]. Based on the NM approach, the ‘parallel chart’ is a tool for collecting the patients’ stories of illness and their interaction of the doctor site, written by physicians. It was implemented in daily medical practice by Rita Charon in 2012 [17] and is a private document in which physicians write also their reflective impressions and emotions towards their patients, without any restrictions [18, 19]. Using this tool, physicians consider the entirety of their patients’ humanity as ‘persons’ who participate in the treatment of their illness and express their goals in their experience [20]. These approaches have been recently discussed by the World Health Organization, which recommended narrative research to improve healthcare quality using a value-based approach [21]. Furthermore, we have recently highlighted in past NM projects applied to the respiratory field, the efficacy of this approach as an educational skill for doctors, and the importance of a trusty relationship with who live with Chronic Obstructive Pulmonary Disease with [22–24].

The main objective of the NM project named SOUND (the Italian acronym of “Writing Narratives on patients with Severe Asthma for a new and Efficacy Diversification and Valorisation of the care”) was to explore RSs’ relationships with severe asthmatic patients in Italy through the language analysis of their narratives. The secondary objective of the project was to understand the factors that influenced doctor-patient interactions and could endanger trust-building.

## Methods

The SOUND project consisted of an educational course followed by a narrative research phase. Eighty-six Italian pulmonologists, allergists, and pediatricians (the ‘RSs’) with expertise in the management of severe asthma were invited to participate in the initiative. Participating physicians first attended a basic training course on writing, analyzing and applying the parallel chart into clinical practice. This course was implemented via webinars held by the ISTUD Foundation in June 2016. The main aim of this training was to transfer the same competence and methods on NM to all RSs in an effort to reduce bias due to personal writing skills.

For the second part of the project, the physicians who took part in the training were invited to write 5 parallel charts each. The parallel chart – designed by a dedicated board consisting of the Healthcare Area of the ISTUD Foundation and three RSs – a pulmonologist, an allergist and a paediatrician [co-authors of this paper] – was structured in prompts that followed a narrative plot (see Appendix 1) that recall the relationship of care from the first re-evaluation of the patient’s clinical condition until today. This semi-structured plot was specifically designed to help physicians overcoming writer’s block [25].

Inclusion criteria for analysis were writing about: a patient with a specific diagnosis of severe asthma; a ≥ 6-month-long doctor-patient relationship; and required that the patient be visited at least twice by the participating RS.

All narratives were collected anonymously from June to November 2016 through a dedicated online platform (https://www.surveygizmo.com/), with no restrictions on the length of narratives.

The study was approved by the ethics committee of the coordinating centre (Calabria Region, Center Area section), and informed written consent was obtained from RSs. All narratives were completely anonymous and RSs were specifically asked not to report any personal, institutional, or geographical information in full compliance with the Declaration of Helsinki.

The parallel charts were analysed according to the Grounded Theory methodology [25]: three researchers independently classified physicians’ narratives to identify recurrent topics [15]. Narratives were also analyzed using special semantic evaluation software, NVIVO 10, which assesses recurrent words and common synonyms to obtain previously unpredictable clusters. The language analysis was carried out using Bury’s classification of narratives [26]. According to this classification, narratives were ‘contingent’ when written very synthetically using a chronological style; they are ‘moral’ when characterized by diffusely moral judgments of the author regarding patients or caregivers; and they are ‘core’ when deeper descriptions of the patients’ illness and greater empathy are evident in the writing. In addition, the emotional examination was carried out using Plutchik’s theory (see Appendix 2) [27] and the emotions described were clustered into 5 main classes: positive emotions, emotions of fear and sadness, emotions of hate and anger, emotions of anticipation, and emotions of submission (that is impotence in facing illness). From observing the used language, three types of doctor–patient relationships were identified: the ‘easy’ and effective relationship wherein there is immediate sympathy between the patient and the physician; the ‘difficult’ one wherein the doctor feels the interaction with the patient as stressing; and the ‘evolved’ relationship that starts with difficulty but improves over time [24]. Descriptive statistics were used to examine the observed type and frequency of language, emotions and, relationships exhibited by RS in writing their parallel charts.

## Results

Sixty-six RSs (77% of the webinar education class) completed the project. The total number of parallel charts collected was 314, an average of 4.7 narratives from each participant. The average time to write each narrative was 36 min and 47 min for adult and pediatric patients, respectively. Basic socio-demographic characteristics of the participants are summarized in Table 1. Adults and children were described in 246 and 68 parallel charts, respectively; however, two parallel charts (one for an adult and one for a pediatric patient) could not be analyzed because of unintelligible. One hundred and eighty-three narratives, 58% of the whole collection, mentioned allergies, allergens or allergy treatments and were clustered as allergy experiences, whereas the other 131 parallel charts (42%) were not considered as allergy since it was not clear whether or not referred to allergic patients.

**Table 1:** Basic socio-demographic characteristics of participating

	Paediatricians (*N* = 13)	Allergists (*N* = 16)	Pulmonologists (*N* = 37)
Gender			
Women	85%	44%	30%
Men	15%	56%	70%
Age			
Mean (range), in years	48 (35 to 61)	45 (34 to 59)	48 (27 to 65)
Mean years (range) as physician	21 (9 to 37)	21 (5 to 33)	21 (2 to 39)
Geographical Origin			
Northern Italy	62%	50%	41%
Central Italy	15%	0	24%
Southern Italy and Islands	23%	50%	35%

### Classification of the narratives and doctor-patient relationships

At the beginning of the written text in parallel charts, doctor–adult-patient relationships were ‘easy’ in 57% of cases, and difficult in 43% of cases, while RSs and pediatric patients had an initially problematic interaction in 36% of cases. Consistent with Bury’s classification of narratives, 57% of the parallel charts about adults were considered ‘core’ narratives, whereas 37% ‘contingent’, due to the brevity and poverty of the emotions described therein. The remaining 6% were ‘moral’ narratives since they were written in a judgmental fashion towards patients and their families. Differently, comparing the physicians’ style of writing, the ‘moral’ style rose up to 19% of cases in narratives about children and adolescents, while ‘core’ and ‘contingent’ decreased to 48 and 33%, respectively (Table 2).

**Table 2:** Classification of narratives

	Adult patients (*N* = 246)	Paediatric patients (*N* = 68)	
		Children/Adolescents	Caregivers
Relationships at the beginning			
Easy relationships	57%	64%	57%
Difficult relationships	43%	36%	13%
Relationships at the end			
Easy relationships	57%	64%	57%
Difficult relationships	9%	11%	13%
Evolved relationships	34%	25%	30%
Bury’s classification			
Core narratives	57%	48%	
Moral narratives	6%	19%	
Contingent narratives	37%	33%	

### Factors influencing doctor–patient relationships at the beginning of the narratives

The initial adult patients’ emotions most frequently mentioned by RSs overall were fear (27%), submission (21%) and sadness (18%). The remaining narratives described patients with positive feelings such as joy, trust, and optimism (11%) or negative feelings such as anger and aggression (8%). These emotions directly influenced doctor–patient relationship, as well as physicians’ emotional status. In particular, difficult adult interactions were featured by a higher presence of patient’s anger (96% higher in ‘core’, 55% in ‘contingent’, and 80% in ‘moral’ narratives), compared with easy relationships, in which the prevalent emotion were fear-and-sadness and positivity (Table 3). Submission was present in narratives describing both difficult and easy relationships with adult patients. A particular pattern emerging from the narratives was that the relationship was easier and more effective when RSs took into account patients’ fear of the illness symptoms (Table 3). Another emotion felt by physicians in easy relationships with adult patients was mainly anticipation, since they were waiting to know how illness might have developed (Table 4). In contrast, in difficult doctor–adult-patient relationships, RSs often felt anger towards their patients’ feelings of resignation, because they wanted patients actively react to symptoms and improve their engagement in the care. Risk factors which most affected doctor–adult-patient relationships were: obesity, smoking, online research, and homeopathic therapies. Comorbidities, having pets, divorces, or grief were not found to influence the effectiveness of RSs’ interactions with asthmatic adults (see Appendix 3).

**Table 3:** Factors influencing doctor–patient relationships at the beginning of the narratives

	Core Narratives		Contingent Narratives		Moral Narratives	
	Easy	Difficult	Easy	Difficult	Easy	Difficult
Emotions at the first visit (Adult patients)	(*N* = 79)	(*N* = 57)	(*N* = 38)	(*N* = 31)	(*N* = 3)	(*N* = 10)
Fear and sadness	52%	44%	45%	45%	33%	30%
*‘The patient seemed depressed […] since he was particularly hindered by his symptoms in daily activities’.* *‘The patient was afraid and worried about the future since he was able to assess the severity of his disease through the high number of positive prick tests’.*						
Submission	20%	28%	3%	32%	33%	20%
*‘The patient told me it was still difficult for her not to consider herself as a sick person, at life-threatening risk’.* *‘“Doc, they’ve tried everything, you’re not going to tell me that there are further drugs to try, are you? I’ve been sick for 10 years!” I tried to explain to her that new drugs really exist...’*						
Positive	11%	2%	37%	0%	0%	20%
*‘At every visit, he appeared me calm and relaxed, not revealing any sort of fear or worry’.* *‘The patient appeared to be aware of the gravity of his health status, but calm. He was serene when he told me about his allergy and the worsening of its symptoms’.*						
Hate and anger	1%	17%	8%	16%	0%	30%
*‘During the visit, the patient was upset, with a defiant attitude’.* *‘The patient appeared to be tired, uninspired and angry with his situation and clinical condition’.*						
Anticipation	16%	9%	8%	6%	33%	2%
*‘I think that the patient cheered himself up, then asked me for more information about the new therapeutic options’.* *‘During the communication of re-evaluation results, I think the patient felt hope for the possibility of trying a new therapy’.*						
Emotions at the first visit (Paediatric patients)	(*N* = 26)	(*N* = 13)	(*N* = 7)	(*N* = 6)	(*N* = 5)	(*N* = 4)
Fear and sadness	46%	46%	57%	0%	40%	75%
*‘The patient seemed sad, without dreams or hope. He explained his health status in a detached manner’.* *‘In a sense, he felt different from other children because of asthma – he could not play soccer, the most interesting thing to him – so he viewed himself as the “ugly duckling”’.*						
Submission	4%	0%	0%	33%	0%	0%
*‘When I saw this little girl under a beach umbrella near mine, she stayed still, while other guys, her cousins, were playing and moving around her’.* *‘The patient seemed to me to be quite passive, of few words – he had half-opened eyelids and a half-opened mouth’.*						
Positive	19%	8%	43%	0%	40%	0%
*‘That day, they entered my clinic smiling and quite happy to see me’.* *‘At her arrival at the clinic, she was smiling and miming a sort of embrace’.*						
Hate and anger	8%	38%	0%	50%	0%	25%
*‘The boy was uncomfortable and anxious to exit from the room and stop our visit’.* *‘His eyes were looking to me as if he didn’t want to be there at that moment’.*						
Anticipation	23%	8%	0%	17%	20%	0%
*‘In the afternoon he visited me to certify his ability to participate in sports, at which he really wanted to do well; however, he told me to be calm since he was feeling good at that period of time’.* *‘The little girl child was sawing through me with her big black eyes’.*						
Emotions at the first visit (RSs in regard to adult patients)	(*N* = 72)	(*N* = 6)	(*N* = 50)	(*N* = 39)	(*N* = 3)	(*N* = 10)
Fear and sadness	17%	19%	9%	24%	0%	11%
*‘I felt powerless and afraid. She couldn’t survive alone in such a severe condition, not only of her illness but of her family situation’.* *‘So, I felt despondent… she was not even allergic, so I could not even use my best therapeutic options’.*						
Submission	1%	2%	0%	6%	0%	0%
*‘Actually, when the patient has a relapse or when unusual symptoms had occurred, he will be more anxious and afraid for his health status, so he uses to go on visit each time he wants, without prior notice’*						
Positive	17%	4%	20%	0%	0%	11%
*‘The patient told me to be calm, so I reflected his emotions’.* *‘I felt good – I was encouraged by the patient since, although I was asking her to come back very often to the hospital for treatment, she appeared to be happy’.*						
Hate and anger	0%	14%	3%	35%	0%	44%
*‘I felt angry and powerless when I tried to convince her to take care of herself’.* *‘I saw for the umpteenth time that the patient had not taken any of the treatment I had prescribed, in spite of severe asthma he suffered from’.*						
Anticipation	65%	61%	69%	35%	100%	33%
*‘He felt hope in regard to his treatment as if it could restore his serenity and calmness. He wished his girlfriend could get a good treatment for the symptoms she had. So I felt I was complicit with the couple in restoring the everyday energy they had in the past’.*						
Emotions at the first visit (RSs in regard to children)	(*N* = 23)	(*N* = 11)	(*N* = 6)	(*N* = 6)	(*N* = 5)	(*N* = 4)
Fear and sadness	9%	7%	0%	0%	0%	0%
‘*I was sad because her mother was crying’.*						
Submission	0%	0%	0%	0%	0%	0%
*Not Applicable (N/A)*						
Positive	9%	0%	33%	0%	40%	25%
*‘The child was listening to me with interest, so she asked: “Can you cure me?” I smiled at her, answering that surely I would try with her parents to make her feel better’.*						
Hatred and anger	17%	43%	33%	33%	0%	50%
*‘The patient seemed like a* “*bully”*, like an uncaring guy who only wants to appear sure of himself’. *‘The re-evaluation visit was very poor since neither the patient nor his family were aware of the privilege they had if they would accept a new, effective, and expensive therapy!!’*						
Anticipation	65%	50%	33%	67%	60%	25%
*‘His family hoped I could have found a solution for the suffering of their child. So I felt a great responsibility towards them’.* *‘I left the patient with one of my colleagues… Coming back home, I was tired but happy knowing the patient was feeling better. I reflected myself and tried to understand if the patient worsened because he was not adherent or because physicians hadn’t followed him in the correct way... doubts that I would like to clarify the following day’.*

**Table 4:** Factors influencing doctor–patient relationships at the end of the narratives

	Core Narratives			Contingent Narratives			Moral Narratives		
	Easy	Difficult	Evolved	Easy	Difficult	Evolved	Easy	Difficult	Evolved
Emotions at the end (Adult patients)	(*N* = 77)	(*N* = 4)	(*N* = 51)	(*N* = 39)	(*N* = 4)	(*N* = 17)	(*N* = 3)	(*N* = 5)	(*N* = 5)
Fear and sadness	3%	25%	2%	0%	25%	0%	0%	20%	20%
*‘Today this person remains on high doses of cortisone and her illness remains uncontrolled. The burden of anxiety and depression on her shoulders worsens the situation’.*									
Submission	1%	25%	2%	0%	50%	0%	0%	20%	0%
*‘Although doctors were giving hope and suggesting possible therapeutic solutions to her, symptoms continued in an increasingly more aggressive way. She could not live or breathe. Any kind of therapy could console her’.*									
Positive	94%	25%	94%	100%	0%	88%	67%	20%	80%
*‘He told me he had written on Facebook, without reference to any person, “My breathing is restored, I can smell perfumes”’.* *‘Today, this person is more serene and active. The asthma treatment allowed him to control his life, his actions, and not to put limits on his living because of his illness’.*									
Hate and anger	0%	0%	0%	0%	25%	0%	0%	40%	0%
*‘I haven’t had news about this patient for several months’.*									
Anticipation	3%	25%	2%	0%	0%	12%	33%	0%	0%
*‘Today this person lives with his illness with more courage and dedication, and also tries to live a mostly normal life, being aware that his condition could worsen at any moment’.*									
Emotions at the end (Paediatric patients)	(*N* = 24)	(*N* = 2)	(*N* = 11)	(*N* = 7)	(*N* = 2)	(N = 3)	(*N* = 5)	(*N* = 2)	(*N* = 2)
Fear and sadness	0%	0%	9%	0%	0%	0%	0%	0%	0%
‘*The situation has not gotten better. They told me they would wait before deciding to start a new therapy. They were afraid’.*									
Submission	0%	0%	0%	14%	50%	0%	0%	0%	0%
*‘He was feeling better, but his parents didn’t allow him to do anything’.* *‘On that occasion, I had managed to re-establish contact with the patient and gain their esteem and confidence, but actually, I was not totally sure. Indeed, at the first visit after release from the hospital, her father accompanied her punctually, but he never returned for follow-up visits’.*									
Positive	96%	50%	73%	86%	0%	100%	100%	50%	100%
‘*He was not obligated to use the bronchodilator continuously anymore. He said, “I used to not care about it, but now it is a new and different life for me! Now I can see and feel the difference!”’* *‘She ran towards me and embraced and kissed me. She said that thanks to me she could realise her dream to swim’.*									
Hatred and anger	0%	50%	9%	0%	50%	0%	0%	50%	0%
*‘Her dream was dancing, but she was continuously tired, more often than other girls, and she felt shame because of her problem with her weight’*									
Anticipation	4%	0%	9%	0%	0%	0%	0%	0%	0%
*‘The little girl appeared calm and she seemed to have accepted both the exams and therapies that will follow’*									
Emotions at the end (RSs in regard to adult patients)	(*N* = 72)	(*N* = 6)	(*N* = 50)	(*N* = 39)	(*N* = 3)	(*N* = 10)	(N = 2)	(*N* = 3)	(*N* = 6)
Fear and sadness	4%	33%	2%	0%	0%	0%	0%	0%	0%
*‘The therapy for asthma, in her case, was not successful and it didn’t allow her a normal life or the tranquillity to raise her children well’.* *‘She usually came back to my center and now has accepted treatment, but I don’t think she has yet to accept the psychological burden of her illness’.*									
Submission	0%	33%	0%	0%	0%	0%	0%	0%	0%
*‘From the relationship I had with this patient, I have learned that acceptance of their illness could be less dramatic than the illness itself’.*									
Positive	4%	0%	86%	51%	0%	80%	0%	0%	33%
*‘And I thought that the therapy would be effective overall!!’* *‘I have reached this goal: the happiness of a man who had met me having past failures ’.*									
Hate and anger	1%	1%	0%	3%	67%	0%	50%	67%	33%
*‘In this case, biological therapy, although efficacious, was not possible due to patient anxiety, probably amplified by the patient’s family situation and by unreliable data found online’.* *‘I was not sad knowing she would not return and I thought that anyone could be nice to everyone in this world, and this was one of those kinds of cases’.*									
Anticipation	0%	33%	0%	0%	0%	0%	0%	0%	0%
*‘I decided, at the third visit, to accept her in a clinical study for a new therapy (double blind versus placebo), and she accepted despite several kilometres that she has to do each time, and despite she knows she could receive placebo…’*									
Emotions at the end (RSs in regard to paediatric patients)	(*N* = 23)	(*N* = 3)	(*N* = 11)	(*N* = 5)	(*N* = 3)	(N = 3)	(*N* = 5)	(*N* = 2)	(*N* = 2)
Fear and sadness	0%	50%	0%	17%	0%	0%	0%	50%	0%
*‘The patient appeared me quite calm, but less than his usual because in that afternoon he would have the examination and the medical visit for having the Suitability to sport. He plays tennis and he loves it’*									
Submission	0%	0%	0%	0%	33%	0%	0%	0%	0%
*‘I thought something could had changed for this child thanks to benefiting of the therapy, but his parents were too overprotective and anxious’*									
Positive	87%	50%	100%	83%	0%	100%	100%	50%	100%
*‘I arranged with her mother to go to the swimming pool to surprise my patient. Immediately she ran to embrace me, doing the “peace sign” with the hands. Her face emanated joy, and I was incredibly touched by seeing her swimming’.*									
Hate and anger	9%	0%	0%	0%	67%	0%	0%	0%	0%
*‘I thought that perhaps what I had been doing would be useless since the patient and his family seemed to be indifferent’.* *‘I thought that the boy’s health was more important than anything else, so his mother must better organise her time since the dates for injections had been communicated to them in time!!’*									
Anticipation	0%	0%	0%	0%	0%	0%	0%	0%	0%
*Not Applicable (N/A)*									

Results for children’s and adolescent’s emotions were quite similar to those for adult patients. Fear and sadness were the most frequent children’s emotions in easy relationships, whereas anticipation was the predominant physicians’ emotion towards pediatric patients (Table 3). In addition to that observed for adult patients, risk factors that influenced paediatric relationships were ‘totally absent’ or ‘hyper-protective’ families and prior hospitalizations, particularly in moral narratives (see Appendix 3).

### Factors influencing the evolution of the doctor–patient relationship

Thirty-four percent of doctor–adult-patient relationships had evolved from a difficult to an easy relationship whereas only 9% remained difficult. In the paediatric context, 11% of difficult doctor–child-patient relationships remained problematic at the end. The highest percentage of initially difficult stories that evolved into positive ones were recorded in ‘core’ narratives (92% of ‘core’, 79% of ‘contingent’, and 69% of ‘moral’ narratives). During the progression of the parallel charts, adult patients’ emotions, as fear, sadness, submission, and anger were recorded only in those narratives where doctor–patient interactions were still difficult at the end, whereas in easy and evolved relationships emotions were mainly positive (Table 4). In the end, in ‘moral’ narratives wherein adult patients and RSs were on bad terms, physicians’ emotions declared were anger, up to hate. However, in difficult relationships between doctor and paediatric patient or children’s caregiver, anger was reported at the end of parallel charts from children and from their relatives, whereas this negative emotion was less present overall from physicians (Table 4).

Although it was not required, 82% of all the RSs’ parallel charts mentioned the therapies prescribed to their patients. The most commonly reported treatments were anti-immunoglobulin E (anti-IgE or ‘biological therapy’) and generic terms as ‘new therapies’, ‘innovative therapies’ for the treatment of allergic asthma, and ICS-LABA for the treatment of intrinsic asthma. In general, physicians proposed to switch the therapy to 83% of adult patients with extrinsic asthma; however, in 6% of the parallel charts, patients’ discontinuation of treatment or disapproval of the therapy switch by the patient, with no shared decision making, was reported. RSs wrote, with no explicit prompts, that their patients considered positively the efficacy of new therapies (61% for adults and 46% for children). In 60% of adult cases, this kind of therapies was not proposed by the physician, and it was not clear whether it happened because the latter opted for other treatments or because they had doubts on dealing with such a challenging procedure with a person they were not on good term with. To exploring the role of the relationship in the improvement of patients’ QoL, a cross analysis between the type of language, the kind of interaction and the acceptance of switch therapy was carried out (see Table 5)*.* The percentage of adult patient’s activity restored were lower when the relation remained difficult than in those cases of positivity at the end (43% of ‘core’, 33% of ‘contingent’ and 57% of ‘moral’ narratives). The patient’s acceptance of a new therapy was more frequently described in ‘core’ narratives where the doctor- adult patient relationships ended easily (58 and 52% of cases begun positively and evolved, respectively), while ‘contingent’ writings were associated with no change therapy proposal, especially where the relation had not an easy beginning (67 and 55% of cases begun negatively and evolved, respectively). Conversely, the highest percentage of new therapies refusal was associated to relations that remained difficult (43% of ‘core’, 17% of ‘contingent’ and 14% of ‘moral’ narratives).

**Table 5:** Acceptance of therapy switch to anti-IgE in relation to the quality of the established doctor–patient relationship

	Core Narratives			Contingent Narratives			Moral Narratives		
	Easy	Difficult	Evolved	Easy	Difficult	Evolved	Easy	Difficult	Evolved
Adult patients	(*N* = 73)	(*N* = 7)	(*N* = 50)	(*N* = 52	(*N* = 6)	(*N* = 22)	(*N* = 10)	(*N* = 7)	(*N* = 8)
Activities restored	79%	43%	84%	58%	33%	45%	70%	57%	75%
New therapy acceptance	58%	29%	52%	33%	17%	45%	80%	14%	75%
New therapy refusal	11%	43%	10%	15%	17%	0%	10%	14%	13%
No change therapy proposal	32%	29%	38%	52%	67%	55%	10%	71%	13%
Paediatric patients	(*N* = 31)	(*N* = 3)	(*N* = 11)	(*N* = 7)	(*N* = 3)	(*N* = 3)	(*N* = 5)	(*N* = 2)	(*N* = 2)
Activities restored	97%	33%	100%	71%	33%	100%	100%	50%	100%
New therapy acceptance	39%	33%	9%	43%	33%	67%	40%	50%	0%
New therapy refusal	23%	33%	55%	29%	0%	0%	20%	50%	0%
No change therapy proposal	39%	33%	36%	29%	67%	33%	40%	0%	100%

Most physicians concluded their parallel charts by explaining what they had learned by applying NM to their clinical practice (90% of all parallel charts collected). In particular, in the majority of cases, participants described the main positive perceived benefits of NM using words and phrases such as ‘empathy’, ‘courage’, ‘positivity’ and ‘importance of the family’, and they explicated that NM could have a key role in the improvement of trust in doctor–patient relationships (93% for adult patients and 78% for paediatric patients). In other narratives, RSs reported the importance of NM towards improving clinical aspects of asthma treatment (7% about adult and 22% about paediatric patients, respectively). The NVIVO analysis identified important words for differentiating difficult relationships from those evolved ones, including ‘courage’, ‘perseverance’ and ‘thoughtfulness’, which were used less frequently in narratives of problematic relationships (20% of difficult and 46% of positive relations at the end, respectively).

## Discussion

The SOUND project is the first Italian NM-based research applied to the severe asthma field. From literature, people living with a chronic disease, such as severe asthma, are at greater risk for uncontrolled symptoms and recurrent hospitalizations [28], hence, there is a need of patients’ engagement in the care and trust in their physicians [10–14]. Furthermore, the interest of the scientific community on the deeply understanding not only of the patients’ needs but also of the caregivers’ role, is actually increasing in the care of respiratory chronic diseases [29]. According to participants, comprehension, interest, and anticipation were the main lessons learned after experiencing both the NM training course and the application of the narrative approach in their clinical practice. In particular, RSs reported that active listening enabled a deeper understanding of motivations that lead the person to evaluate and encounter new lifestyles and therapies.

Results from this project revealed that physicians involved in the care of severe asthma are generally able to manage their emotions, establishing good relations with their patients. In several cases, however, doctors’ anger remained difficult to manage, as confirmed by a recent study on self-reported physicians’ emotions [30]. According to our results, the main reason for being angry with adult patients were the patients’ distrust in prescribed therapies and a bad life styling as smocking or obesity, while in paediatrics the problematic relationships were caused by parental excess of anxiety or completely inattention towards children’s symptoms, leading to physicians’ ‘moral’ style and judgement. These negative feelings of hate and anger, indeed, had often impaired patients’ trust, which is desirable since it was shown in the literature that the establishment of a trusting relationship fosters the likelihood of adherence to prescribed drugs and control visits [13, 14, 30]. Further, when the relation is difficult, RSs proposed less frequently a change of therapy compared to easy ones. This tendency could be linked to the prescription of biological therapies that are considered challenging because of their high costs and the requirement of several subcutaneous injections performed in the hospital [31]. Other narratives from this project revealed in details that patients who refused a change therapy were not able to perform their daily activities and hobbies as they wished, so the relation remained difficult. Consequently, the ‘moral’ attitude and the prejudice on patients and caregivers is useless in establishing effective care. A recent NM educational project held in the respiratory field, indeed, highlighted the association between the physicians’ style of writing, according to Bury’s theory, and the attitude to doctor-patient communication. [24]. In particular, the empathic listening (‘core’ attitude) was often described by physicians as a way to earn the patients’ trust towards their options for better lifestyles and innovative treatments, confirming the importance of finding out relational strategies to reach efficacy and trusty encounter.

The unilateral point of view (from RSs) of the study is a limit of the SOUND project design; only physicians were involved in the initiative, with no written narratives of patients and caregivers. The emotional status of patients and their families were described and reported by RSs; therefore, patients’ emotions might have been misunderstood by their physicians. Consequently, the inclusion of patients’ and caregivers’ listening has to be considered the main objective for future NM projects applied to severe asthma, as recently published in other NM projects on chronic obstructive pulmonary disease [22, 23].

## Conclusion

The SOUND project revealed that the NM approach and application of its parallel chart tool can be effective for helping physicians to better understand and more deeply analyse their own relationships with patients and caregivers. Although for many physicians it may be difficult to think of reflective writing as part of daily clinical practice [32], its introduction could be very useful to promote empathy in difficult cases for instance when the physician does not feel any spontaneous sympathy for the patient and their family [33]. Best practices in doctor-patient communication were diffusely investigated in the respiratory field [34, 35] but, at the moment, no studies have deeply examined the specific role of caregivers’ emotions in the establishment of effective relationships, despite the recognized importance of the family in the care of severe asthma [36]. In the present project, the role of caregivers in both asthmatic adults’ and paediatrics’ relation of care with physicians was observed; consequently, the analysis of not only patients’ but also their caregivers’ narratives could be interesting to carry out as a future challenge.

## Data Availability

All the data of the project are described in the submitted paper. The original narratives, in Italian, are available on the website www.medicinanarrativa.eu.

## Appendix 1

**Table 6:** Prompts of the parallel chart

The visit in which I made the revaluation of the patient was […]. The patient seemed to me […]. While, his/her relative […]. The patient told me (refer especially to the emotional, familiar, of working and of activities spheres) […]. While his/her relative told me […]. Consequently, I do […]. During the communication of revaluation results, I thought that patient was feeling […]. While his/her relative was feeling […]. So, I was feeling […]. I thought that […]. And I […]. During the following visit/s […]. The patient told me (refer especially to the emotional, familiar, of working and of activities spheres) […]. And his/her relative […]. The patient’s activities […]. I thought that […]. And I […]. Today, this person […]. The asthma care […]. From the relationship with this patient and his/her family I have learned that […]. For the future I would like I […]. For the future I would like for him/her […]. Thanks for the time, the energy, and for the dedication you spent for us. We would like to propose you just another question: How do you have felt in writing the parallel chart? […].	

## Appendix 2

**Table 7:** Plutchik’s emotional classification

Less intense emotions	Primary emotions	The most intense emotions
Optimism		
Serenity	Joy	Ecstasy
Love emotions		
Acceptance	Trust	Admiration
Submission		
Apprehension	Fear	Terror
Awe		
Distraction	Surprise	Amazement
Disapproval		
Pensiveness	Sadness	Grief
Remorse		
Boredom	Disgust	Loathing
Contempt		
Annoyance	Anger	Rage
Aggressiveness		
Interest	Anticipation	Vigilance

Plutchik theory (Table 8), emotional analyses methodology: grey boxes are intermediate emotions between the box above and the other one below, that are divided in further three levels of intensity (from less intense to the most one).

## Appendix 3

**Table 8:** Other factors influencing doctor-patient relationships at the beginning of the narratives

Negative factors	Narratives examples
- Obesity	*“L. is a middle-aged lady who’s been suffering from asthma for almost all her life; she’s allergic to dust mites and pollen, she’s always had anxiety and obesity issues. Lately, her anxiety worsened, together with her weight gain”;* *“Metaphor: Dear whale; the patient told me she was completely handicapped in her daily activities. She could not walk without experiencing shortness of breath, so she never went out alone. Immediately, she started crying and said her obesity was the reason for her unhappiness and illness. She had pledged to eat less, to no avail”.*
- Smoking	*“I must confess it was very annoying for me: when they came to see me, I sometimes didn’t even visit F. I used to explain in detail how damaging it was for their daughter to be exposed to their passive smoke and ask them to be careful, because the pharmacological therapy could be compromised by smoke. They justified themselves saying they only smoked on the terrace, and only rarely inside the house, and never in the same room where the kid was. So, they were convinced they were not doing F. any harm”;* *“She continued smoking 2-3 cigarettes per day and she even confessed me she smoked marijuana 3-4 times a week. I tried to discourage her from the habit of smoking, but I haven’t obtained any result. I invited the patient to stop smoking, but the patient refused the very idea”.*
- On-line researches	*“This patient is the kind of person who likes to ask plenty of questions, carefully assessing the pros and cons before making a decision. I thought she was one of those difficult patients who have a list of questions ready each time and do a lot of on-line research, asking me for reassurance on the therapies I had proposed”;* *“The follow-up visit was peculiar; the patient was hasty, she did not want to listen to me, but only to propose something she’d read on-line or on some magazine. The patient seemed to be confused about all the aspects of the matter, because she was too involved in the media”.*
- Homeopathic therapies	*“She immediately said she was against medical treatments, because she preferred homeopathic drugs. Sincerely, I don’t know what this lady was feeling, because she was too distant from me, so it was impossible for me to catch any impression of her. After explaining for 30 minutes what asthma is, how to treat it, what mistakes are not to be done with it, she insisted on me prescribing some homeopathic drugs, so I felt a strong sense of rebellion against it. I thought I had to explain her everything from the beginning once again, but I did not feel like it; in addition, talking with her was really unsatisfying for me”;* *“I explained her that homeopathic drugs could be very dangerous, because they are completely useless in the treatment of pathologies with uncontrolled symptoms, as severe asthma. In addition, using these drugs could increase the risk of recurrent exacerbations and hospitalization (uncontrolled asthma). All the same, the patient continue to apparently agree with me, but just formally, showing she had indeed understood but she was not REALLY aware of what I had just explained, even about the risk of exacerbations if she persisted in using homeopathic drugs that totally exclude the allopathic treatment”.*
- The hyper-protective of pediatric patients	*“I could not understand why no one before me had the child’s health as a priority. In addition, I could not understand why such an anxious and careful mother didn’t take her child to another doctor for a second opinion. The kid seemed particularly low, resigned and a little bit difficult, while his hyper-anxious mother was explaining to me each bronchospasm episode in details. […] His parent was in crisis; his mother was doubtful and terrified by the potential side effects of the biological therapy I proposed. […] I thought they were foolish to reject my proposal to change therapy!”*
- absent families of pediatric patients	*“The patient told me he felt better with the new therapy, while his parents said they were not so attentive with him, so they didn’t show so much enthusiasm about it; … I felt frustration. […] and I thought that he would probably suspend the therapy, because his family did not feel like coming to the hospital so frequently”.*
- Prior hospitalizations of asthmatic children	*“S. and her young mother had a bewildered and fearful look: in their eyes there was the terror of future hospitalizations and other bad experiences. The kid didn’t speak a word, she just sat there, next to her mother. She carefully observed her surroundings, trying to catch just a “sign” in my gestures, in my eyes and in my words. […] On that occasion I tried to reassure them by saying that all these feelings were normal after this kind of ordeal. I told them that we would have had frequent visits over the following months, in order to bring the kid’s asthma under control”.* *“She was just 2 years old: her pediatrician had suggested further examinations, because of her recurrent and often severe episodes of bronchospasm, that in some cases had led to hospitalization. […] Some of her positive attitude rubbed off onto me: I was calm and happy to be part of their family. We were all aware we were going to make it!!”*

## Abbreviations

Anti-IgE: Anti-immunoglobulin E
GINA Guidelines: Global Initiative for Asthma Guidelines
ICS: Inhaled corticosteroids
LABA: Beta-agonists
NM: Narrative Medicine
QOL: Quality of Life
RS: Respiratory Specialists
SOUND project: The Italian acronym of “Writing Narratives on patients with Severe Asthma for a new and Efficacy Diversification and Valorisation of the care”
